# Phase II study of helical tomotherapy in the multidisciplinary treatment of oligometastatic colorectal cancer

**DOI:** 10.1186/1748-717X-7-34

**Published:** 2012-03-16

**Authors:** Benedikt Engels, Thierry Gevaert, Hendrik Everaert, Peter De Coninck, Alexandra Sermeus, Nicolas Christian, Guy Storme, Dirk Verellen, Mark De Ridder

**Affiliations:** 1 Department of Radiation Oncology, UZ Brussel, Vrije Universiteit Brussel, Laarbeeklaan 101, B-1090 Brussels, Belgium; 2 Department of Nuclear Medicine, UZ Brussel, Vrije Universiteit Brussel, Laarbeeklaan 101, B-1090 Brussels, Belgium; 3 Department of Gastroenterology, UZ Brussel, Vrije Universiteit Brussel, Laarbeeklaan 101, B-1090 Brussels, Belgium

**Keywords:** Metastatic colorectal cancer, Oligometastases, Helical tomotherapy, IMRT-IGRT

## Abstract

**Background:**

Complete metastasectomy provides a real chance for long-term survival in patients with oligometastatic colorectal cancer (CRC). For inoperable patients, we evaluated in this study intensity-modulated and image-guided radiotherapy (IMRT-IGRT) by helical tomotherapy.

**Methods:**

Twenty-four CRC patients with ≤ 5 metastases were enrolled, receiving a dose of 50 Gy in fractions of 5 Gy. No limitations concerning dimension or localization of the metastases were imposed. Whole body PET-CT was performed at baseline and 3 months after the initiation of RT to evaluate the metabolic response rate according to PET Response Criteria in Solid Tumors (PERCIST) version 1.0.

**Results:**

A total of 53 metastases were treated. Seventeen patients (71%) received previously ≥ 1 line of chemotherapy for metastatic disease, displaying residual (n = 7) or progressive (n = 10) metabolic active oligometastatic disease at time of inclusion. Most common sites were the lung, liver and lymphnodes. One patient (4%) experienced grade 3 dysphagia. Twenty-two patients were evaluated by post-treatment PET-CT. Twelve patients achieved a complete (n = 6) or partial (n = 6) metabolic response, resulting in an overall metabolic response rate of 55%. At a median follow-up of 10 months, 7 patients (29%) are in remission, of which 5 received previous chemotherapy with residual oligometastatic disease at time of inclusion. The actuarial 1-year local control, progression-free survival, and overall survival were 54%, 14% and 78%.

**Conclusions:**

Helical tomotherapy delivering 10 fractions of 5 Gy resulted in a metabolic response rate of 55%, and appeared to be attractive as consolidation of inoperable oligometastatic disease after effective chemotherapy.

**Trial registration:**

Eudract 2008-008300-40; NCT00807313

## Background

Nearly one fourth of the patients with newly diagnosed colorectal cancer (CRC) present synchronous liver metastases and more than half of the resected CRC patients develop distant recurrence during follow-up [1]. The diagnosis of metastatic CRC (mCRC) does however not equal an acute fatal illness and might be classified more as a chronic disease in patients with a clinical disease state between locoregionally confined and widely spread metastatic disease, so called oligometastatic disease [2,3]. It is well understood today that a complete metastasectomy provides a real chance for long-term survival in those patients, with documented 10-year overall survival (OS) rates in 1 out of 6 mCRC patients which underwent hepatic resection [4]. According to the data of the randomized EORTC intergroup Trial 40983, the perioperative administration of 5-fluorouracil (5-FU) and oxaliplatin (FOLFOX) has emerged as a new standard of care in mCRC patients with limited resectable liver metastases, by increasing the 3-year progression-free survival (PFS) with 8% (36% versus 28% without perioperative chemotherapy) [5]. Despite the improvements in surgical procedures and preoperative multi-agent chemotherapy, limitations imposed by localization, multifocal character, size, or comorbidities still exclude the major part of mCRC patients from undergoing metastasectomy [6]. Aiming at ablating metastases while preserving the surrounding healthy tissues, there has been an expansion over the past decade in the use of non-surgical local ablative alternatives such as radiofrequency ablation (RFA) and stereotactic body radiotherapy (SBRT), the latter a tailored delivery of tumoricidal doses of radiation in a minimal number of fractions to small lesions by the combination of high conformal RT and rigorous localization of the target by image-guided RT (IGRT) [7-10]. In the eradication of liver- and lungmetastases by SBRT, limited toxicity rates and sustained local control (LC) are reported by several authors up [7,8]. However, in order to allow delivery of those cytotoxic doses, SBRT requires carefully selection of the metastases on the base of their localization and dimension. Our institution previously explored in a pilot study the efficacy and toxicity of helical tomotherapy, a technology combining rotational intensity-modulated RT (IMRT) and IGRT by megavoltage (MV) computed tomography (CT) scanning, in patients with oligometastatic CRC who were not amenable for metastasectomy and (further) systemic therapy [9]. In order to suit a variety of treatment sites including large metastases and critically located lesions, we used a moderately hypofractionated regimen, delivering 10 fractions of 4 Gy. This schedule resulted in a complete metabolic response (CMR), the primary objective, in 5 out of 23 enrolled oligometastatic CRC patients [9]. Besides, helical tomotherapy displayed a very safe toxicity profile, with grade 2 and 3 toxicity recorded in only 9% and 4% of the patients, respectively [9]. Taking into account the rather disappointing 1-year LC rate of 54% in this pilot study, we now aimed for higher response and LC rates in those patients. To do so, we investigated in this study the efficacy and toxicity of helical tomotherapy delivering 50 Gy in daily fractions of 5 Gy in patients with inoperable oligometastatic CRC. The primary objective was to evaluate the CMR rate 3 months after initiation of RT by performing whole-body ^18 ^F-fluorodeoxyglucose (FDG)-positron emission tomography (PET) at baseline and at evaluation. Secondary endpoints were toxicity, LC and PFS.

## Methods

### Patient population

Patients with a radically resected primary tumor with the histological proof of a colorectal adenocarcinoma and at time of inclusion 1 to 5 metastases, showing increased metabolism on ^18^FDG-PET, were eligible for this study. No limitations were imposed on the localization or dimension of the metastases. Patients were required to have an Eastern Cooperative Oncology Group performance status of ≤ 2, to be > 18 years old and inoperable by the localization, number or dimension of the metastases, medically unfit to undergo resection or refusing surgery. Patients were not permitted to receive chemotherapy within 1 month before initiation of RT. Patients who did not receive previous chemotherapy for metastatic disease had to be medically unfit to undergo systemic treatment or refusing chemotherapy. Patients with Child B or C liver cirrhosis or a functional liver volume < 1000 cc in case of liver metastases and a lung diffusion capacity for carbon monoxide (DLCO) of < 30% in case of lung metastases were excluded. Patients with an active second primary tumor were excluded. All patients signed study-specific informed consent. The protocol was reviewed and approved by the Ethical Committee and registered (inter)nationally (Eudract 2008-008300-40; NCT00807313).

### Pre-treatment evaluation and radiotherapy technique

Pre-treatment evaluation included a complete medical history, physical examination, a pre-treatment free breathing ^18^FDG-PET and computed tomography (CT) using a dedicated PET-CT camera (Gemini TF, Philips Medical Systems, OH, USA) and laboratory tests including carcinoembryonic antigen (CEA) with assessment of the Child-Pugh parameters and liver enzymes in patients with liver metastases. RT was performed using the TomoTherapy Hi⋅Art II System (TomoTherapy Inc., Madison, WI), which fully integrates IGRT by means of MVCT scanning and IMRT by means of dynamic rotational therapy [10]. The gross tumor volume (GTV) included the visible gross tumor mass on CT. The GTV was expanded by a 10, 10 and 12 mm for the anteroposterior, laterolateral and craniocaudal direction, respectively, to create the planning target volume (PTV), fully encompassing the ^18^FDG-PET-positive volume. The planning goals were to deliver at least 95% of the prescribed dose to at least 95% of the PTVs, while keeping the maximum dose (D_max_) to the PTV below 105%. The volume of lung receiving more than 20 Gy (V_20_) was kept below 20% in case of lung irradiation. In patients with liver metastases, the liver volume receiving more than 22 Gy (V_22_) and 30 Gy (V_30_) was kept to less than 50% and 30%, respectively. A D_max _of 36 Gy (72% of the prescribed dose) was set to the spinal cord. In case of intersection between the PTV and hollow viscous organs (small bowel, large bowel, stomach or oesophagus) of ≥ 5 cc, a PTV subvolume was defined at this interface with a D_max _of 40 Gy (80% of the prescribed dose) to this overlap volume. The treatment was delivered daily in 10 fractions, excluding weekends. Before each treatment session, patients underwent scanning using the integrated MV-CT scan modality and were repositioned after co-registration of these images with the planning kilovoltage (kV)-CT scan.

### Toxicity monitoring

Toxicity was evaluated and scored according to the National Cancer Institute Common Terminology Criteria for Adverse Events (NCI CTC AE) version 3.0, with toxicity occurring within 3 months after the initiation of RT classified as acute toxicity. Patients were contacted and/or invited for follow-up 3-monthly during the first year, 6-monthly thereafter.

### Treatment evaluation

Response evaluation has been described extensively in the previous pilot study in our institution [9]. Briefly, the primary objective, CMR rate, was evaluated by comparing the PET-CT at baseline with the PET-CT performed 3 months after initiation of RT, according to the PET Response Criteria in Solid Tumors (PERCIST) version 1.0 [11]. A CMR in a patient was defined by a complete resolution of ^18^FDG uptake within all irradiated lesion(s), so that it is less than mean liver activity and indistinguishable from surrounding background blood-pool levels, without new ^18^FDG-avid lesions in pattern typical of CRC. For a partial metabolic response (PMR), a reduction in SUV_max _for patients with one lesion or a reduction of the sum of the SUV_max _data for patients with > 1 lesion of minimum 30% was required, without the appearance of new ^18^FDG-avid lesions. Obvious progression of any lesion (> 30% increase in SUV_max_) or new ^18^FDG-avid lesions negate a partial response and indicate progressive metabolic disease (PMD). Stable metabolic disease (SMD) is defined as not CMR, PMR, or PMD.

During follow-up, the Response Evaluation Criteria in Solid Tumors (RECIST) were used to evaluate response. A local recurrence was defined as the re-growth of tumor within or at the periphery of the irradiated volume. The appearance of new lesions was considered as distant recurrence.

### Statistics

A Richard Simon two-stage optimal design was performed to obtain the sample size. Aiming at an overall acceptable and unacceptable CMR probability of 50% and 30%, respectively, with an α and β value of 0.10, the sample size for first and second stage were 7/22 and 17/46 evaluated patients, respectively. Actuarial LC, PFS and OS rates were estimated by Kaplan-Meier analysis, Log-rank testing was used to evaluate the association between patient-related factors and treatment outcome. Laboratory tests were evaluated by *t *tests.

## Results

### Patient characteristics

Twenty-four inoperable oligometastatic CRC patients with a total number of 53 metastases were enrolled between March 2010 and July 2011. Patient characteristics are given in Table 1. Seventeen patients (71%) received previously ≥ 1 line of chemotherapy for metastatic disease, of which 7 and 10 patients presenting residual and progressive metabolic active oligometastatic disease at time of inclusion, respectively. Seven patients (29%) received no previous chemotherapy for treatment of metastatic disease; 5 patients were medically unfit to undergo systemic therapy and 2 patients refused chemotherapy.

**Table 1:** Patient characteristics (n = 24)

Variable	Distribution	No. of Patients	%
Sex	Male	14	58
	Female	10	42
Age (years)	Median	67 years	
	Range	45 - 91 years	
Karnofsky Performance status	Median	90	
	Range	50 - 100	
Previous chemotherapy	0	7	29
(number of lines)	1	3	13
	2	10	42
	3	2	8
	4	2	8
Previous local therapy	No	7	29
for metastases	Yes	17	71
Number of metastases	1	10	42
	2	5	22
	3	4	16
	4	4	16
	5	1	4
Gross tumor volume (cc)	Median	7 cc	
	Range	1 - 100 cc	
Number of involved sites	1	17	71
	2	4	17
	3	3	12
Localization	Liver	7	29
	Lymph node	9	38
	Lung	13	54
	Peritoneum	2	8
Follow-up (months)	Median	10 months	
	Range	3 - 21 months	

Seventeen patients (71%) received previous local therapy for metastatic disease; radiotherapy (n = 7), RFA (n = 6) and/or metastasectomy (n = 8). Twelve patients (50%) presented with a GTV located in the vicinity of hollow viscous organs (small/large bowel, stomach or esophagus).

### Toxicity

All patients finished their RT course without interruptions of toxicity reasons. Grade 3 acute adverse events were observed in 1 patient (4%), which displayed grade 3 dysphagia due to irradiation of an infracarinal lymph node metastasis. No other grade ≥ 3 acute side effects occurred. Two patients (8%) and 1 patient (4%) experienced grade 2 dysphagia and diarrhea after irradiation of mediastinal and pelvic lymph node metastases, respectively. Of the 14 patients that were irradiated for lung and/or mediastinal lymph node metastases, 5 patients (36%) and 4 patients (29%) displayed grade 1 pneumonitis (asymptomatic, radiographic findings only) and grade 2 pneumonitis (symptomatic, not interfering with activities of daily living), respectively. The recorded average V_20 _of the lung for the 5 patients without pneumonitis was 6.9% ± 4.1% compared to 15.7% ± 8.6% for patients with grade ≥ 1 pneumonitis (n = 9) (p = 0.06). Average functional liver volume (liver - GTV) for the patients irradiated for liver metastases (n = 7) was 1412 cc ± 265 cc. No radiation-induced liver disease (RILD) was observed in those 7 patients. With recorded average V_22 _and V_30 _of the liver of 27.0% ± 12.7% and 16.0% ± 7.5%, respectively, no violation of the liver dose-volume constraints occurred. At a median follow up of 10 months (range, 3 - 21 months), 1 patient (4%) that was irradiated on 4 lymph node metastases in the coeliac and liver hilar region developed grade 2 pyloric ulcera 7 months after the end of RT. The dose to the nearby stomach and duodenum was limited to maximal 4 Gy/fraction in this patient. Endoscopic biopsy in this patient suggested the presence of gastric antral vascular ectasia. No grade 3 late adverse events occurred until now.

### Response evaluation

**Figure 1:**
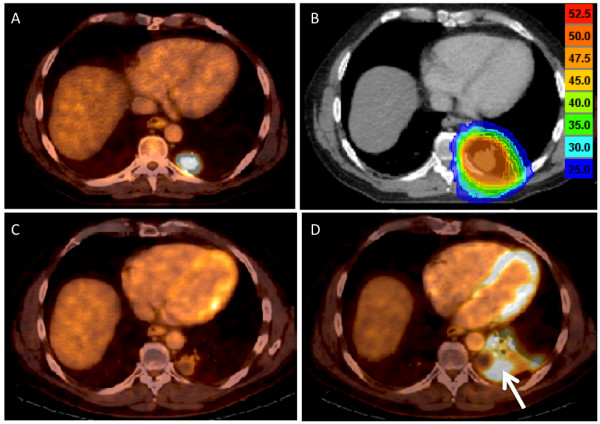
**Complete metabolic response displayed in a colorectal cancer patient with a solitary lung metastasis**. (**A**) Pre-treatment ^18 ^F-fluorodeoxyglucose (FDG)-positron emission computed tomography (PET-CT) of a metastatic colorectal cancer patient with a solitary metabolic active lungmetastasis. (**B**) Planning CT with superimposed radiation dose distribution. (**C**) Complete metabolic remission on PET-CT scan 3 months after initiation of radiotherapy. (**D**) PET-CT performed 6 months after completion of radiotherapy shows no evidence of progressive disease, but note the occurrence of an asymptomatic radiation induced pneumonitis around the irradiated metastasis (white arrow).

Two out of 24 enrolled patients failed to receive a PET-CT 3 months after the initiation of RT, 1 because of poor general condition and 1 being evaluated in another institution after the end of treatment. Six patients (27.3%) achieved a complete metabolic response (CMR), 6 patients (27.3%) a partial metabolic response (PMR), resulting in an overall metabolic response rate of 55%. The mean fractional change in SUV_max _at evaluation as compared to baseline was -56.1% (range, 43.5% - 70.2%) and -40.2% (range, 33.3% - 45.1%) for complete and partial metabolic responders, respectively. Figure 1 illustrates a CMR in a patient treated for lungmetastasis. Metabolic response rates are listed in Table 2. Seven patients (32%) displayed progressive metabolic disease (PMD), of which 6 patients at distance and 1 patient both in- and outfield. Three patients (13%) presented stable metabolic disease (SMD). Regarding the 17 patients who received previously ≥ 1 line of chemotherapy, a metabolic response was recorded in 86% (n = 5) of the patients with residual metabolic active disease at time of inclusion, compared to a 40% (n = 4) metabolic response rate in the patients with progressive metabolic active disease before RT. Lastly, 50% (n = 3) of the evaluated patients who did not receive previous chemotherapy for treatment of metastatic disease displayed a metabolic response 3 months after initiation of RT. Monitoring of the tumor marker CEA at baseline showed a mean value of 8.8 ± 8.7 ug/L for the whole patient group, with elevated CEA (> 3 ug/L) recorded in 71% (n = 14) of the patients. A significant decrease of the CEA level was recorded in patients with a CMR or PMR (n = 12), with a mean value of 7.7 ± 8.6 ug/L pretreatment compared to 4.6 ± 6.6 ug/L 3 months after initiation of RT (p = 0.01). Metabolic non-responders (SMD or PMD, n = 10) displayed a pretreatment CEA level of 10.5 ± 9.5 ug/L, compared to a posttreatment value of 27.5 ± 41.6 ug/L (p = 0.18).

**Table 2:** Metabolic response rate 3 months after start of radiotherapy by ^18 ^F-fluorodeoxyglucose (FDG)-positron emission tomography (PET) (n = 22 patients)

	No. of patients	%
Complete metabolic response	6	27.3
Partial metabolic response	6	27.3
Stable metabolic disease	3	13.6
Progressive metabolic disease	7	31.8
No PET	2	

### Follow-up

**Figure 2:**
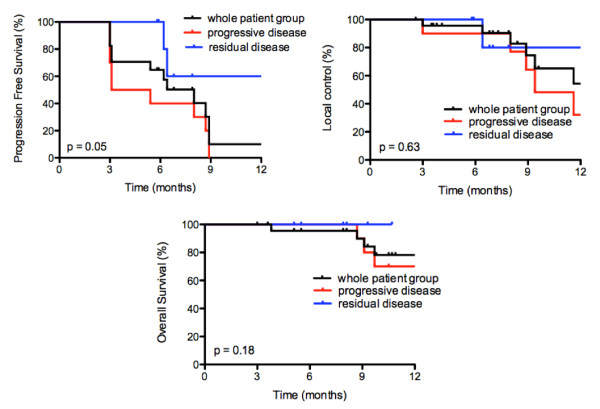
**Local control (LC), progression-free survival (PFS) and overall survival (OS) rates among the whole patient group, previous systemically treated patients displaying residual metabolic active oligometastatic disease at time of inclusion (n = 7) and patients with progressive metabolic active disease before RT (n = 10)**. Log-rank testing was used to evaluate the association between response on previous chemotherapy and treatment outcome, with p values reported.

With a median follow-up of 10 months (range, 3 - 21 months), 7 patients (29%) are in remission in all irradiated areas without evidence of distant recurrence. Five patients died, 4 because of progressive metastatic disease and 1 because of non-cancer related cerebral bleeding. Seventeen patients (71%) developed progressive disease, of which 10 patients distant recurrence, 6 patients synchronous local and distant progression, and 1 patient with isolated local recurrence, the latter underwent wedge resection of a solitary progressive lung metastasis. Among the other 16 patients with progressive disease, 5 patients received best supportive care (BSC), 6 patients systemic therapy, 4 patients an additional course of RT (2 patients because of distant relapse and 2 patients for local and distant relapse), 1 patient underwent metastasectomy of a new livermetastasis. We report a 1-year actuarial LC, PFS and OS of 54% (95% C.I. 23-78%), 14% (95% C.I. 3-35%) and 78% (95% C.I. 52-91%), respectively. Log-rank testing found a statistically significant benefit of the post-treatment outcome in terms of PFS for patients with a metabolic response (CMR + PMR) at 3 months after RT as compared with metabolic non-responders (SMD + PMD) (p < 0.01). For the patients who received previous chemotherapy (n = 17), log-rank testing revealed that patients with residual disease pre-RT exhibited a trend toward superior PFS (p = 0.05) compared with patients presenting progressive disease at time of inclusion, whereas LC and OS were not significantly different (p = 0.63 and p = 0.18, respectively) [Figure 2]. Of the 7 patients which were in remission at a median follow-up of 10 months, 5 had residual oligometastatic metabolic active disease after previous chemotherapy at time of inclusion. Factors such as number of metastatic lesions, number of involved sites or number of previous lines of chemotherapy were not correlated with post-treatment outcome.

## Discussion

Laboratory and clinical data recently supported the hypothesis that oligometastatic disease is a distinct clinical entity, where tumors early in the evolution of metastatic progression produce metastases limited in number and location [12-14]. The potential temporal evolution with the intermediate stage of limited metastatic capacity, where oligometastatic tumors may not have acquired the broad array of genetic changes to develop widespread metastases, is strongly suggested by novel insights into the metastatic evolution of pancreatic cancer [12,14]. Yachida and Campbell et al. discovered that the clonal populations which give rise to distant metastases were genetically evolved from the original non-metastatic clone, with a required time period of at least 5 year before full acquisition of metastatic ability [12,14]. The proposed hierarchal character of metastatic progression in time and number in those landmark investigations emphasizes the need of aggressive treatment of oligometastases, which may affect cure rates in a selected proportion of metastasized patients [13]. Especially, the treatment of oligometastatic CRC remains an attractive area of investigation as CRC patients tend to develop metastases limited in number and location. It is well accepted that complete resection of CRC liver metastases provides a real chance for long-term survival, with reported 5-year OS rates of 30-40% [15,16]. Despite the improvements in surgical procedures and preoperative multi-agent chemotherapy, limitations imposed by localization, multifocal character, size, or comorbidities still exclude the major part of mCRC patients from undergoing metastasectomy [17,18]. Recent evolutions in conformal RT and IGRT have driven the development of SBRT, of which the physical properties allow sparing of the surrounding normal tissues with consequently fewer side effects to be expected [7,10]. Next, it enables delivery of tumoricidal doses of radiation in a minimal number of fractions to small target volumes. In the eradication of CRC liver- and/or lungmetastases, minimal toxicity and sustained LC rates of 53%-100% are reported with the use of SBRT [8,19,20]. However, SBRT requires a careful selection of the metastases on the base of their localization and dimension, as patients with metastases situated in the proximity of hollow viscous organs such as small bowel, esophagus or stomach could experience unacceptable normal tissue toxicity with the delivery of ablative radiation doses by SBRT [21]. In order to suit a variety of treatment sites, we explored in a previous study in our institution the use of moderately hypofractionated IMRT-IGRT (10 fractions of 4 Gy) by helical tomotherapy in oligometastatic CRC [9]. Taking into account the very limited toxicity (4% grade 3 toxicity) and the relatively high local progression (22%) of irradiated metastases without development of new metastases in this trial, the aim was on higher response rates and LC. In this report, we present the results of a prospective phase II trial of helical tomotherapy delivering 50 Gy in daily fractions of 5 Gy to inoperable oligometastatic CRC patients. The primary objective was to evaluate the CMR rate by comparing ^18^FDG-PET 3 months after initiation of RT with ^18^FDG-PET at baseline. A sample size for first stage of 7/22 was calculated based on a Richard Simon two-stage optimal design aiming at an overall acceptable and unacceptable CMR probability of 50% and 30%, respectively. After having evaluated 22 patients by ^18^FDG-PET, a CMR was documented in 6 patients. As 10 fractions of 5 Gy showed lower than expected activity in first stage (6/22), the second stage of accrual is not carried out and the trial has been terminated. Indeed, the overall metabolic response rate of 55% at 3 months and 1-year LC rate of 54% are similar to the rates observed after 10 fractions of 4 Gy in our previous study [9]. In comparison, Milano et al. reported with 50 Gy in 10 fractions in oligometastatic patients a 2-year LC rate of 67% [22]. One should bear in mind that LC rates of more than 95% with SBRT are reported with biologically effective doses (BED) of > 100 Gy, which can be only safely delivered in patients ideally with maximal 3 metastases of less than 4 cm in diameter and far from hollow viscous organs. Although not superior to 10 fractions of 4 Gy, the delivery of 50 Gy (BED of 75 Gy assuming an α/β of 10 for tumor response, corresponding to a biologically equivalent total dose in 2-Gy fractions (EQD_2Gy_) of 62.5 Gy) with IMRT-IGRT by helical tomotherapy in a non-selected patient population resulted in a promising overall response rate of 55%, which is higher than the response rates achieved with second- and third-line systemic treatment regimens in mCRC, which are within the range of 9-37% [3,23].

Of notice, we enrolled in our previous study mCRC patients who already received previous systemic treatment only in the case when they presented progressive disease or cumulative toxicity limiting further continuation of systemic treatment [9]. Actually, not only patients with progressive disease or cumulative toxicity limiting further continuation of systemic treatment were enrolled in the current study, but also mCRC patients with residual metabolic active oligometastatic disease after effective previous systemic treatment (n = 7). The use of helical tomotherapy as consolidation appeared to be highly attractive in this subgroup, reflected by a 86% metabolic response rate in those patients, and a trend toward increased PFS (p = 0.05) as compared to the patients with no response on previous systemic treatment. At a median follow-up of 10 months, 71% of those patients (n = 5) are still in remission in all irradiated areas without evidence of distant progression, whereas 4 of the 10 patients presenting progressive disease after previous chemotherapy at time of inclusion already died because of progressive metastatic disease. To our knowledge, these findings for the first time indicate a potential role for the use of RT as consolidation of previous systemically treated oligometastatic CRC. This creates opportunities for future trials of SBRT that should tailor inoperable oligometastases according to their previous response to systemic treatment, finally to resolve its value in this patient population which is historically considered to be incurable when treated with chemotherapy alone. Only in abstract form, Ruers et al. recently suggested as first a potential benefit for combining systemic and local treatment in mCRC by presenting the results of a randomized phase II study which evaluated the benefit of RFA combined with chemotherapy compared to chemotherapy alone in 119 mCRC patients with unresectable liver metastases [24]. Although a statistically significant benefit in median PFS has been reported for the RFA + chemotherapy arm (16.8 months versus 9.9 months for patients receiving chemotherapy alone, p = 0.03), the follow-up and study design (primary endpoint: 30-months OS > 30%) do not allow a formal comparison between the 2 treatment arms in terms of OS [24]. Lastly, in concordance with our previous experience, a metabolic response (CMR or PMR) 3 months after initiation of RT was also found to be predictive in terms of time to progression (p < 0.01). Hence, ^18^FDG-PET should be offered complementary to anatomical imaging for all oligometastatic CRC patients undergoing a RT course.

The delivery of 50 Gy in 10 fractions by the combination of dose sculpting by IMRT with image-guidance techniques by the Tomotherapy Hi-Art II System appeared to be a safe regimen, with grade 3 acute and late toxicity recorded in only 1 patient. The lowering of the maximal dose to 4 Gy/fraction on the stomach and duodenum did not prevent the occurrence of grade 2 pyloric ulcera in the patient treated because of 4 perihilar lymph node metastases. Taking into account the radiosensitive nature of the stomach and duodenum, patients with metastases located in the proximity of those organs should be excluded from high-dose SBRT. The limited toxicity in the present study in a patient population with critically located lesions supports the further use of a moderately hypofractionated RT regimen, such as 10 × 5 Gy.

Finally, from a technical point of view, the IGRT solution in the Tomotherapy Hi-Art II system allows only pre-treatment management of tumor motion by volumetric imaging (MV-CT) and thus requires the application of CTV to PTV margins in the order of 1 cm to account for intrafraction tumor motion, for example in the lung and liver. Theoretically, real-time tracking of the metastases during treatment should allow a strong reduction of the CTV to PTV margin, and thus less healthy tissues need to be irradiated. This is especially attractive in performing dose escalation in critically located lesions, as classical CTV to PTV margins result in significant overlap between PTV and organs at risk, the latter limiting delivery of cytotoxic doses with regard to normal tissue toxicity. The VERO system is a novel platform for image-guided SBRT designed to anticipate tumor motion during treatment by real-time tracking of the target [25]. Its dynamic capabilities have been explored currently in our department and a clinical trial investigating its value in the eradication of inoperable oligometastases will be initiated. In the context of dose escalation, one should also mention the potential of particle therapy, being proton therapy the example, in minimizing the irradiated volume of the surrounding healthy tissues compared to 3D conformal RT and IMRT [26].

## Conclusions

In conclusion, 10 fractions of 5 Gy resulted in a promising metabolic response rate of 55% and limited toxicity. Helical tomotherapy may further play a substantial role in the multidisciplinary treatment of inoperable oligometastatic CRC, especially as consolidation in patients with residual oligometastatic disease after being treated systemically.

## Competing interests

The authors declare that they have no competing interests.

## Authors' contributions

Concept and design: BE, HE, GS, DV, MDR. Acquisition, analysis and interpretation of data: BE, TG, HE, PDC, AS, NC, MDR. Drafting of the manuscript: BE, MDR. Reading and approval of final manuscript: all authors.
